# An Overview of Lung Cancer in Women and the Impact of Estrogen in Lung Carcinogenesis and Lung Cancer Treatment

**DOI:** 10.3389/fmed.2021.600121

**Published:** 2021-05-17

**Authors:** Vianey Rodriguez-Lara, Maria Rosa Avila-Costa

**Affiliations:** ^1^Department of Cell and Tissue Biology, Faculty of Medicine, Universidad Nacional Autonoma de Mexico, Mexico City, Mexico; ^2^Neuromorphology Laboratory, Facultad de Estudios Superiores Iztacala, Universidad Nacional Autonoma de Mexico, Mexico City, Mexico

**Keywords:** lung cancer, women, estrogens, antiestrogen medication, adenocarcimoma

## Abstract

Lung cancer incidence and mortality have significantly increased in women worldwide. Lung adenocarcinoma is the most common form of lung cancer globally. This type of lung cancer shows differences by sex, including the mutational burden, behavior, clinical characteristics, and response to treatment. The effect of sex on lung cancer patients' survival is still controversial; however, lung adenocarcinoma is considered a different disease in women and men. Moreover, lung adenocarcinoma is strongly influenced by estrogen and is also different depending on the hormonal status of the patient. Young pre-menopausal women have been explored as an independent group. They presented in more advanced stages at diagnosis, exhibited more aggressive tumors, and showed poor survival compared to men and post-menopausal women, supporting the role of sex hormones in this pathology. Several reports indicate the estrogen's role in lung carcinogenesis and tumor progression. Thus, there are currently some clinical trials testing the efficacy of antihormonal therapy in lung cancer treatment. This mini review shows the updated data about lung cancer in women, its characteristics, the etiological factors that influence carcinogenesis, and the critical role of estrogen in lung cancer and treatment.

## Introduction

Lung cancer in women (LCW) is a severe health problem globally. Smoking habits remain the main factor for its development; however, a high percentage of women with lung cancer (LC) are non-smokers. Therefore, the studies should focus on identifying the risk factors for LCW besides tobacco smoking. Recently, it has been reported that sex and sex hormones may influence LC behavior, survival, and treatment response.

### LCW Epidemiology

LCW has significantly increased worldwide in the last years, while in men gradually has decreased, in women has exceeded the incidence and mortality reported for breast cancer, being the leading cause of cancer death in developed countries such as the United States and some from Europe (1–5). Nowadays, LCW has reached third place in incidence and the second place in mortality worldwide with an estimated of 725,352 cases and 576,060 deaths in 2018 (6, 7).

By 2030, LCW is expected to increase 43% in 52 countries with a median of LC age-standardized mortality rates (ASMRs) rising from 11.2 to 16, which will exceed the ASMRs observed for breast cancer (8). Additionally, the current smoking prevalence in adolescent girls from low and middle-income countries of Africa, South America, and the Middle East is higher than that observed in many high-income countries (9), which will increase LCW incidence and mortality in the following years if smoking is not reduced.

LCW increase, mainly in developed countries, could be explained partially by the high tobacco intake (5–9). However, LC has also increased in non-smokers and young women worldwide. Moreover, only 50% of LCW is associated with tobacco smoke. Recently, Jemal and coworkers reported that the increased LC incidence in young women in the USA is not fully explained by smoking patterns (10). Other factors, in addition to tobacco smoking, are essential to developing LCW.

### Characteristics of LCW

According to the histological features, two types of LC have been described: small-cell lung cancer and non-small cell lung cancer (NSCLC). Currently, the most common type of LC worldwide is the NSCLC, being 85% of all LC diagnosed. There are three NSCLC subtypes: squamous cell carcinoma, lung adenocarcinoma, and large cell carcinoma. Approximately 40 to 60% of LCW correspond to adenocarcinoma, while squamous cell carcinoma is about 10 to 30% of all LC diagnosed (11–13). Lung adenocarcinoma is a heterogeneous type of cancer and exhibits a lesser association with smoking habits compared to other LC subtypes. It is estimated that up to 50% of women with lung adenocarcinoma are non-smokers, compared to 10–15% of non-smoking men who develop this type of cancer (14).

Clinical characteristics are different between women and men; the diagnosis age in women is lower, women more often have a non-smoking habit, present less advanced stages at diagnosis, and the outcome and survival are significantly better at all stages (11). Hormonal status is rarely considered in studies; however, when pre-menopausal women have been studied independently, they were more commonly diagnosed in advanced stages, exhibited less differentiated tumors, and showed a higher number of metastases and poor prognosis compared to post-menopausal women and men (15–18). It was recently reported that pre-menopausal women exhibit lower overall survival than men and post-menopausal women, supporting the role of sex hormones in LC progression (19).

It is also noteworthy that women with NSCLC respond differently to treatments, being more responsive to chemotherapy (mainly platinum-based treatment), those with adenocarcinoma histology type (13), and for the therapy-based on tyrosine kinase inhibitors (TKIs), but less sensitive to immunotherapy (20–22).

Some LC mutations are more frequent in women, and some genes are differentially expressed by sex. The cytochrome CYP1A1 is overexpressed in women (23), partially explaining tobacco carcinogens' highest susceptibility, since CYP1A1 bioactivates these compounds. Women have a reduced DNA repair capacity (23) and increased gastrin-releasing peptide receptor (GRPR) that stimulate cancer cell proliferation. Tobacco-related p53 mutations are also more frequent in women (24, 25). All these data suggest that women are more susceptible to tobacco carcinogens than men.

Moreover, women are more likely to be ERCC1-negative, which could explain the chemotherapy treatment benefit compared to men. The Kristen rat sarcoma virus (KRAS) and epidermal growth factor receptor (EGFR) are often overexpressed in women's lung adenocarcinoma (25). Mutations in EGFR (exon 18–21) are more frequent in women and are associated with estrogen receptor expression (25–28).

### LCW Etiological Factors

Tobacco smoking remains an essential factor associated with LCW, being more susceptible and having more risk of developing LC than men even in similar smoking exposure (29). The increased incidence of LCW in developed countries has been associated with the changes in smoking habit; however, LCW has risen in young women (30) and even in non-smokers (31), supporting the importance other factors have in lung carcinogenesis in women.

Secondhand smoke is a relevant risk factor in non-smoker women; 64% of deaths due to LC associated with secondhand smoke correspond to women. The passive smoker is exposed to two sources of carcinogens: the smoke generated by the cigarette combustion and the smoker's smoke exhales. The benzo-a-pyrene diol epoxide, a primary carcinogen of tobacco, is found in both sources (32).

Wood smoke exposure (WSE) affects women mainly in developing countries, where wood is used for cooking and keeping the home warm. For example, it is estimated that 34.4% of non-smoking patients with LC in Mexico were chronically exposed to wood smoke. WSE is associated with adenocarcinoma subtype and higher EGFR mutation (33). WSE produces an increase in MMP-2 and MMP-9, DNA breaks strand, and adducts (34). Deregulation in pathways such as PI3K/AKT, MEK/ERK, and genes involved in DNA repair, cell cycle, apoptosis, and vesicle transport have also been observed in WSE patients with LC (35).

Among non-smokers women, who develop LC in China and India, cooking oil fumes is an important risk factor for developing LC (36). When cooking, oil is brought to high temperatures and generates fumes that contain lung carcinogens, such as polycyclic aromatic hydrocarbons, which produce DNA-oxidative damage and lipid peroxidation (37).

Ambient air pollution, mainly particulate matter (PM2.5), is also associated with a high risk for LC (38), being that the hydrocarbons and heavy metals in the PM compounds carry carcinogenic potential. PM exposure produces inflammation associated with LC (39). Some reports have found that women exhibit a higher risk to air pollution than men (40–42), probably due to their higher susceptibility to carcinogens, reduced DNA repair capacity, and polymorphisms of xenobiotic-metabolizing genes. Ambient air pollution remains a significant risk factor for developing LC in non-smoking women who live in cities with high air pollution levels (43).

Previous reports indicated that the human papillomavirus (HPV) infection could be associated with LCW, since DNA from HPV16, 18, 30, 31, 33, and 39 were detected in women LC tissues (44–46); however, recent studies rule out this association, since there is no evidence of DNA from HPV in lung tumors, and no differences were found between the presence of HVP in lung tumors and controls (47–49).

Although secondhand smoke, wood combustion, cooking oil fumes, and air pollution are other risk factors to develop LC, particularly in non-smoking women, this does not fully explain the higher LC incidence because a considerable percentage of women were not exposed to these factors. Therefore, it is necessary to identify other etiological factors associated with LCW since up to 50% of cases exhibit non-smoking association.

### Estrogen Role in Lung Carcinogenesis

Estrogens (E2), through their receptors (ER), regulate several biologic events in addition to their reproductive function. Nuclear estrogen receptor alpha (ERα) and beta (ERβ) are expressed in lung tissue from women and men, playing a role in lung development and physiology (50). The estrogen pathway has also been related to lung carcinogenesis (15).

#### Estrogen Receptors and Aromatase Expression in NSCLC

ER expression has been detected in tumors from patients and NSCLC cell lines (51) and is overexpressed, mainly in lung adenocarcinoma. The ERβ is the most abundant form of ER in LC; it is overexpressed in 60–80% of tumors from women and men (52), and it is related to mutations in ~500 genes (53).

ERβ expression is associated with hormonal status. The highest expression was found in tumors from pre-menopausal women; lower expression was observed in post-menopausal women. The minimum expression was observed in men (16), suggesting the critical role of circulating estrogen in ER expression.

The G protein-coupled estrogen receptor (GPER) has also been identified in the cytoplasm of NSCLC tumors from women and men, showing higher expression and activity than normal bronchial epithelium (54). GPER expression was associated with LC IIIA and IV stages, lymphoid node metastases, and poorly differentiated tumors. *In vitro*, estrogen increased cell proliferation, migration, and invasion through GPER (55).

Aromatase enzyme (ARO) has been detected in NSCLC cell lines and ~86% of tumors. Through ARO, the tumor produces estrogen and activates the estrogen pathway; consequently, it is stimulated by circulating and locally produced estrogen (56). Metastases sites exhibit higher ARO expression than the primary tumor; thus, local estrogen production might also induce the metastases in LC (57).

#### Exogenous Estrogen Exposure and LC Risk

Exogenous estrogen intake and LC risk have also been investigated; however, the results are still controversial. Several studies have shown that hormonal replacement therapy (HRT) is associated with decreased risk of LC and may have a protective effect on the development of NSCLC in women (58, 59). In contrast, some studies report that HRT increases LC incidence (60, 61). A prospective study involved 36,588 women who use combined HRT estrogen-based plus progestin showed 50% of increased risk after ten or more years of treatment (60). Moreover, the women's health initiative studies showed that combined HRT was unrelated to LC incidence. Still, a relation to increased mortality from LC, less differentiated tumors, and distant metastases were observed (62). Poor survival in women who use HRT before cancer diagnosis was found compared to women who never use it (39 vs. 79 months) (63). LC risk decreased after HRT cessation (61).

Regarding contraceptive intake, studies found no association with LC increased incidence (64); however, Iversen and coworkers (65) reported an increased risk among ever users who have smoked. The risk of contraceptive intake once LC is developed has not been investigated.

Another exposure form to exogenous estrogen is through endocrine disruptors such as Bisphenol-A (BPA), promoting the migration and invasion of LC cells (66). It was recently reported in the Chinese population that BPA levels were significantly higher in NSCLC patients than in healthy controls; therefore, BPA exposure may be an important risk factor (67).

Estrogen intake does not appear to increase the LC risk; however, once the disease is established, it may increase mortality, probably due to the carcinogenic mechanisms activated by the estrogen pathway.

#### Estrogen Pathway in Lung Carcinogenesis

Estrogen metabolites generate reactive oxygen species (ROS) that cause DNA oxidative damage and form adducts by associating directly to DNA, causing mutations (52). Moreover, through the genomic pathway, the E2/ER complex promotes NSCLC cell proliferation and cell cycle progression by inducing the estrogen expression-dependent genes, c-myc, cyclin D, and Id proteins genes (68, 69). By the non-genomic pathway, E2 activates several pathways that sustain cell proliferation and stimulate tumor growth, such as AMPc, PI3K, MAPK, AKT, and ERK (69, 70).

The EGFR pathway is one of the most important signaling pathways in NSCLC since 89% of patients exhibit EGFR overexpression or mutation (71). In NSCLC, E2 activates the EGFR pathway even in the absence of its ligand, promoting cell proliferation, survival, angiogenesis, cell migration, and metastases (72). ERβ expression has also been associated with EGFR mutations (73).

Additionally, estrogen induces angiogenesis through vascular endothelial growth factor (VEGF-A), the ligand of VEGFR-2 expressed in endothelial cells. VEGFR activation produces endothelial cell proliferation to form new vessels that support tumor growth (68).

*In vitro*, E2 stimulated CXCR4 expression and CXCL12/CXCR4 pathway activation in a time and dose-dependent manner, resulting in cell migration (74). This pathway also supports cell proliferation, survival, apoptosis resistance, and angiogenesis and stimulates migration and metastases to the brain, bone, liver, and lymph nodes, the main metastases sites of NSCLC. CXCR4 activation is also related to chemoresistance, maintenance of stem cell characteristics in tumor cells, and immunoresistance by recruiting regulatory T cells (Tregs) to the tumor microenvironment (75, 76).

Estrogen modulates the immune response by modifying the tumor microenvironment, stimulating pro-inflammatory cytokines, recruiting Tregs, and promoting cell migration (77). Estrogen induces VEGF secretion by tumor-associated macrophages (TAMs), which support an immunosuppressive tumor microenvironment. ERα expression in lung adenocarcinoma is also associated with Treg recruitment and immunosuppressive response (78).

**Figure 1:**
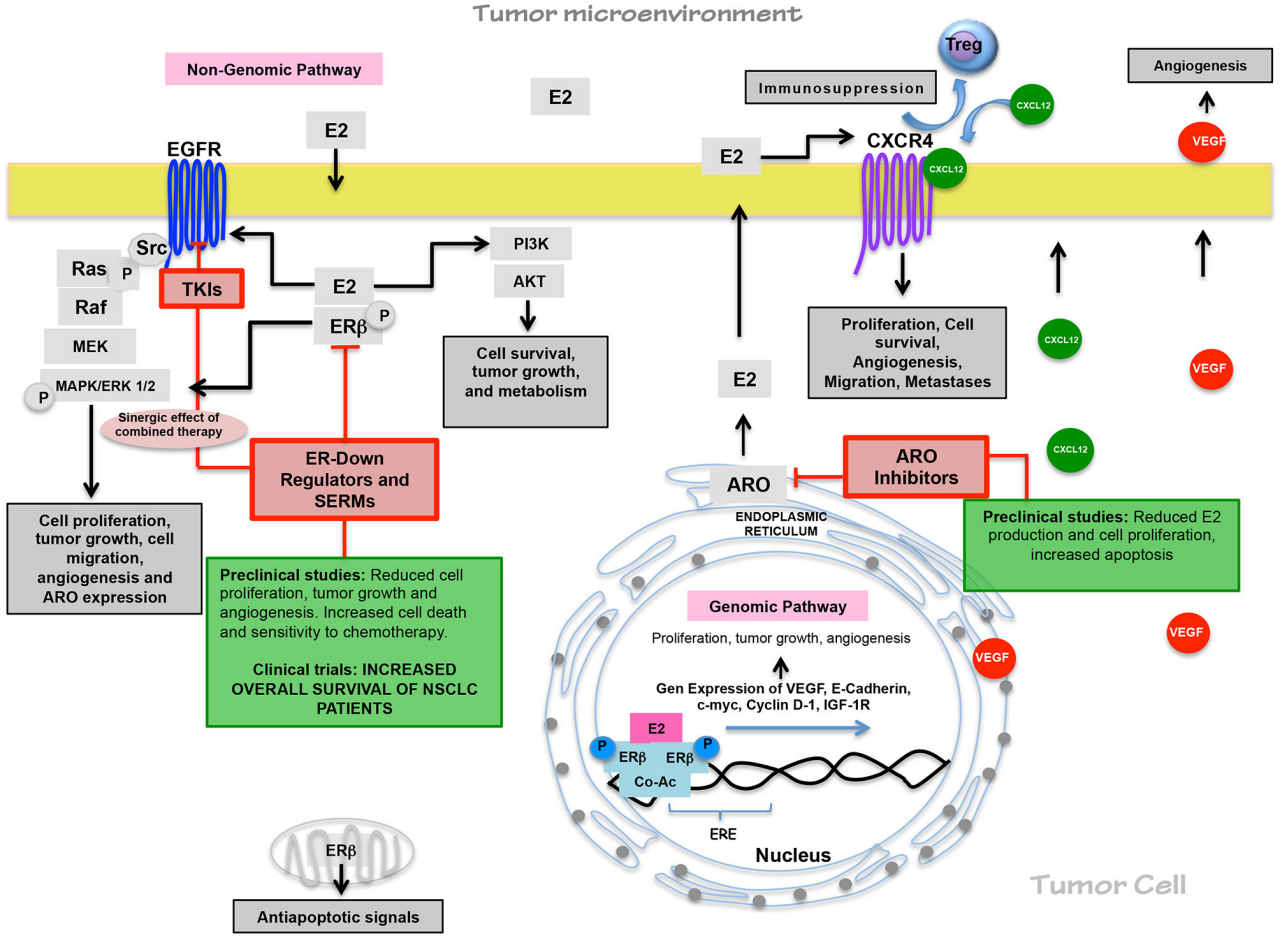
Estrogen pathway in lung carcinogenesis and its relevance on therapy. Through non-genomic and genomic pathways, estrogen promotes cell proliferation, tumor growth, angiogenesis, cell migration, immunosuppressive responses, and tumor progression since tumor cells overexpressed ERβ. ARO expression maintains elevated E2 levels. The functional relationship between EGFR/EGF and E2/ER pathways sustain the E2 production through ARO expression and the EGFR activation in EGF absence. Preclinical studies have shown antiestrogen treatment efficacy, and in clinical trials, antiestrogen drugs have improved the clinical benefits of TKI and chemotherapy.

Nowadays, we know that the estrogen pathway has an essential role in NSCLC, mainly in lung adenocarcinoma, promoting several cancer hallmarks, including cell proliferation, apoptosis resistance, angiogenesis, tumor cell migration and metastases, and probably immune evasion. However, further studies are necessary to fully understand this hormone's relationship with LC progression (Figure 1).

### Estrogen Pathway Relevance as a Target in LC Treatment

The antiestrogen drug's efficacy has been tested in *in-vitro* and *in-vivo* assays in NSCLC. Aromatase inhibitors, such as letrozole, significantly decrease cell proliferation, while exemestane reduces tumor growth, increases cell apoptosis, and inhibits cell migration and invasion *in vitro* (79). The treatment with the estrogen receptor inhibitor, fulvestrant, decreases the tumor growth, the ERβ expression, and the mesenchymal features induced by E2 also increases the chemotherapy sensitivity and T lymphocytes activity (80).

The use of antiestrogen drugs was retrospectively investigated. A higher survival was reported in women who used this therapy previously to be diagnosed with NSCLC than women who never used antiestrogenic treatment (1.89 years vs. 0.93 years, respectively) (81). Also, in clinical trial phases I and II, tamoxifen increased the cisplatin toxicity, and the response to combined therapy was higher than chemotherapy (82). Combined treatment with docetaxel/fulvestrant produced a higher response and overall survival in advanced NSCLC patients than individual chemotherapy (83).

Additionally, the effect of combined antiestrogen and TKI therapy has been investigated in NSCLC due to the functional relationship between estrogen and EGFR pathways. *In vitro*, antiestrogen treatment prevents cell proliferation, stimulates apoptosis, reduces tumor growth, and increases the gefitinib sensitivity (84, 85). A phase I clinical trial tested gefitinib plus fulvestrant efficacy. The results showed improved overall survival in NSCLC patients with a mean of 65.5 weeks in those with higher ERβ tumor expression (86). Also, combined therapy (erlotinib/fulvestrant) has been well-tolerated and demonstrates more clinical benefit than TKI monotherapy (87, 88).

Currently, the effectiveness of antiestrogenic therapy on LC is still being investigated. Approximately ten clinical trials study the effects of antiestrogen therapy alone or combined, mainly with TKI, showing clinical benefits in NSCLC patients. These results will be relevant to propose new treatment schemes in LCW patients (Figure 1).

## Discussion

LCW has significantly increased, even in young and non-smoking women worldwide. Thus, it is necessary to explore the risk factors that explain this occurrence in the female population to strengthen prevention strategies.

The estrogen pathway has shown an important role in lung carcinogenesis, tumor progression, response to treatment, and survival. The exogenous estrogen could be an important risk factor in women with LC and those with a high risk of developing this disease due to a high percentage of NSCLC express ER/ARO.

Although some estrogen pathways in lung carcinogenesis have been investigated, further studies are necessary to fully understand its role in this disease and its relationship with different critical pathways blocked in targeted therapy to enhance its clinical benefit and decrease the resistance.

Since NSCLC is a different disease in women than in men, and hormonal status influences the behavior of LC, future studies must include the highest number of women as possible; also, it is essential to separate women by hormonal status since very few studies have evaluated this variable and could provide vital information to improve the treatment in young pre-menopausal women, whose frequently are diluted in most studies and shows the worst prognosis.

Finally, it is essential to propose specific therapeutic schemes in LC treatment that consider the differences in LC behavior in women since women and men are treated similarly, independently of sex and hormonal status. Because of the relevance that the estrogen pathway has in LC, and with the high percentage of ER expression in LC patients (60–80%), antiestrogen therapy would be an important option for LC treatment. ER detection in NSCLC could be considered in the future to propose better treatment options for women; however, more research is needed in this area.

## Author Contributions

All authors listed have made a substantial, direct and intellectual contribution to the work, and approved it for publication.

## Conflict of Interest

The authors declare that the research was conducted in the absence of any commercial or financial relationships that could be construed as a potential conflict of interest.
